# Personalized Approach to the Role of Endoscopic Ultrasound in the Diagnosis and Management of Pancreaticobiliary Malignancies

**DOI:** 10.3390/jpm11030180

**Published:** 2021-03-04

**Authors:** Michael Makar, Eric Zhao, Amy Tyberg

**Affiliations:** 1 Department of Internal Medicine, Rutgers Robert Wood Johnson Medical School, New Brunswick, NJ 08901, USA; mm2908@rwjms.rutgers.edu (M.M.); ez118@rutgers.edu (E.Z.); 2 Division of Gastroenterology & Hepatology, Rutgers Robert Wood Johnson Medical School, New Brunswick, NJ 08901, USA

**Keywords:** endoscopic ultrasound, pancreaticobiliary malignancy, interventional EUS

## Abstract

Pancreaticobiliary malignancies arise from different areas within the pancreas and biliary tree. Endoscopic ultrasound (EUS) is a well-recognized diagnostic and therapeutic modality in the treatment of pancreaticobiliary diseases, and more specifically, pancreaticobiliary malignancies. Traditionally used for diagnostic purposes, EUS plays a critical role in tissue sampling and cancer staging. The emergence of the new field of interventional EUS has allowed EUS to also play a critical role in therapeutic management. Novel interventional EUS procedures such as EUS-guided gastrojejunostomy (EUS-GE), EUS-guided biliary drainage (EUS-BD), and EUS-guided gallbladder drainage (EUS-GLB) can be utilized to treat complications of pancreaticobiliary malignancies such as gastric outlet obstruction, obstructive jaundice, and cholecystitis. In addition, interventional EUS procedures can be utilized for the palliation of unresectable malignancies both for source control with EUS-radiofrequency ablation (EUS-RFA) and for the treatment of abdominal pain refractory to opioid medications with EUS-guided celiac axis neurolysis. However, patient selection remains a critical component in both diagnostic and therapeutic interventions and must be tailored to individual patient wishes, disease pathology, and overall prognosis.

## 1. Introduction

Pancreaticobiliary malignancies arise from different areas within the pancreas and biliary tree. Among all pancreaticobiliary malignancies, pancreatic cancer is the most common. In the United States, approximately 57,600 people are diagnosed with exocrine pancreatic cancer every year. It is the fourth most common cause of cancer-related death in the US and the second most common cause of digestive cancer-related death [1]. Cholangiocarcinoma is another important pancreaticobiliary malignancy, which accounts for about 3% of all gastrointestinal cancers [2]. In the United States, gallbladder cancer has an incidence of 1–2 cases per 100,000 [3]. Primary ampullary carcinomas are most rare, with an incidence of only 3–4 cases per million of population, with its incidence being increased among patients who have familial adenomatous polyposis or Lynch syndrome [4].

Endoscopic ultrasound (EUS) is a well-recognized diagnostic and therapeutic modality in the treatment of pancreaticobiliary diseases, and more specifically, pancreaticobiliary malignancies. Traditionally used for diagnostic purposes, the emergence of the new field of interventional EUS has allowed EUS to also play a critical role in therapeutic management. However, patient selection remains a critical component in both diagnostic and therapeutic interventions and must be tailored to individual patient wishes, disease pathology, and overall prognosis.

## 2. Patient Selection and Diagnostic Approach

Clinical manifestations for patients with any type of pancreaticobiliary malignancy can be similar. Regardless of the specific type of cancer, these patients have the potential to develop abdominal pain, jaundice, nausea, vomiting, anorexia, and/or weight loss. Patients with any constellation of these symptoms, coupled with serologic evidence of liver or biliary injury, warrant further workup with abdominal imaging. In general, imaging options for all pancreaticobiliary malignancies include computerized tomography (CT) and/or magnetic resonance imaging (MRI) or magnetic resonance cholangiopancreatography (MRCP). Endoscopic retrograde cholangiopancreatography (ERCP) is a highly sensitive modality for the visualization of the biliary tree and pancreatic ducts, although it may be less useful for visualizing the gallbladder and thus gallbladder cancer. In addition, due to the risk of pancreatitis from ERCP, it is often reserved for therapeutic purposes only. EUS is also used in diagnosis. Sugiyama et al. reported that EUS was significantly more sensitive (96%) in detecting pancreaticobiliary carcinomas compared to ultrasonography, CT, and angiography [5]. For ampullary tumors, studies have shown that EUS is superior to CT and ultrasound for detection, but similar to ERCP and MRI [6,7,8]. For gallbladder carcinoma, EUS has a reported better diagnostic sensitivity and specificity than ultrasound [9].

Once a diagnosis is suspected on initial imaging, generally the next step in evaluation is to assess disease extent and cancer resectability. Although histology is required to officially confirm a diagnosis, it is not always necessary. Specifically, for patients who are deemed to have resectable cancer and are fit for surgery, pre-operative biopsy may not be needed before surgery. Further details regarding diagnostic biopsy will be discussed in the next section. Determining disease extent and resectability is carried out via tumor staging, and resectability is based on the presence of distant metastases and the invasion of adjacent structures including vasculature. Multiple imaging modalities can be used for tumor staging, including CT, MRI, positron emission tomography (PET) scan, and EUS. Specifically, EUS can be useful for T-staging and N-staging but not M-staging, which typically requires CT or MRI.

For pancreatic cancer, EUS was shown to be superior to CT for the T-staging of smaller tumors, although the opposite may be true for larger tumors [10]. One meta-analysis of 29 studies by Puli et al. attempted to evaluate the accuracy of EUS in detecting pancreatic tumor vascular invasion. They reported a pooled sensitivity of 73% and a pooled specificity of 90% [11]. Furthermore, in regards to N-staging, EUS was shown to be as accurate as helical and multidetector row CT [12]. Other studies have highlighted the value of utilizing multiple modalities. For example, Shami et al. reported that EUS and MRI had marginal correlation in staging pancreatic cancer, and therefore both tests should be performed for accurate staging [13]. It is also important to keep in mind that EUS is operator-dependent. This can ultimately influence its value in staging accuracy. In fact, one study demonstrated that staging accuracy for pancreatic cancer improved after one-hundred cases, suggesting a correlation between accuracy and the number of procedures performed by the operator [14].

EUS can also be a good staging tool for cholangiocarcinoma, particularly for distal bile duct lesions, by visualizing the local extent of the primary tumor and the status of regional lymph nodes. However, it is less sensitive for staging more proximal lesions. Another study showed that EUS is more sensitive and accurate for identifying portal vein invasion in patients with cholangiocarcinoma [15]. Regarding ampullary carcinomas, a meta-analysis of 14 studies reported a sensitivity and specificity of 77% and 78% for staging T1 tumors and 70% and 74% for determining nodal status [16]. Lastly, for gallbladder cancer, EUS has demonstrated to be a useful tool for assessing tumor depth of invasion [17]. Taken as a whole, EUS is an effective tool for T- and N-staging for all pancreaticobiliary malignancies.

Overall, the choice of imaging modality should be based on the type/size/location of the malignancy, the presence of distal metastases, and the expertise available for different modalities. Smaller pancreatic cancers and extra-hepatic cholangiocarcinomas are well visualized on EUS, while intrahepatic cholangiocarcinomas and large pancreatic tumors may be better viewed on MRI and CT, respectively. Additionally, while the determination of vascular invasion is critical for patients in consideration of surgical resection, it is not required for patients with the presence of distal metastases. EUS is valuable in centers with an operator experience in EUS, but may be less valuable in centers with limited operator experience.

## 3. Diagnostic Tests

The next step after the staging of the tumor is to determine whether or not biopsy is needed. Patients who are good surgical candidates and are staged to have resectable disease do not necessarily need a biopsy prior to proceeding with surgery. However, other patients who do not fit into that category may require histologic confirmation to establish the diagnosis. Situations when a biopsy is useful include the following: there is evidence of systemic disease spread, there is local evidence of cancer unresectability, the patient is unfit for surgery, or an alternate diagnosis needs to be excluded. However, even in good surgical candidates with resectable disease, biopsy may be useful in assisting with surgical planning or ruling out differential diagnoses, such as autoimmune pancreatitis or sclerosing cholangitis. If the decision to obtain a biopsy is made, there are two broad types of biopsy methods that can be done—EUS-guided and percutaneous (ultrasound/CT-guided).

### 3.1. EUS vs. Percutaneous Biopsy

**Figure 1:**
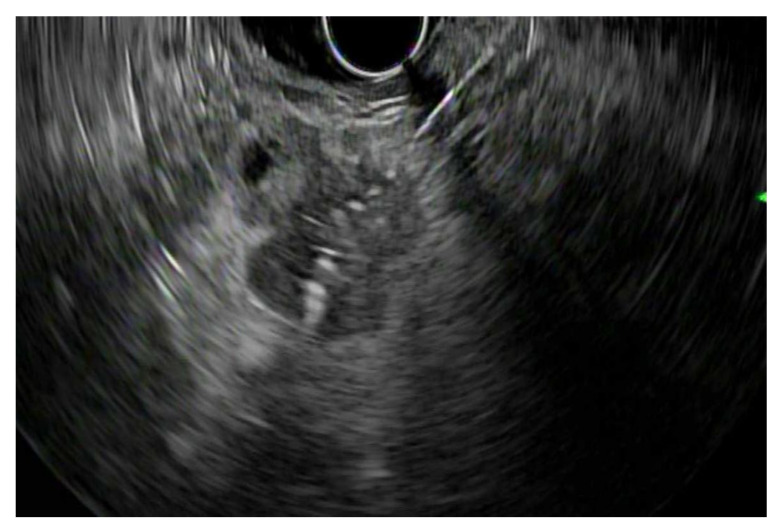
Endoscopic ultrasound-fine needle aspiration (EUS-FNA): Endosonographic image of an EUS-FNA of a pancreatic mass.

EUS-guided biopsy can be accomplished with either fine needle aspiration (FNA) or core needle biopsy (Figure 1). EUS-FNA is performed with an EUS-guided puncture of a lesion followed by the aspiration of cells or fluid for cytology. FNA is often regarded as the best modality for obtaining a tissue diagnosis. It has the capability to biopsy lesions too small to be characterized by CT or MRI, and it can biopsy lesions too close to vascular structures for safe percutaneous biopsy. EUS-FNA can also biopsy small metastases in the left liver lobe as well as diagnose carcinomatosis via ascitic fluid sampling [18,19]. EUS-FNA generally has good sensitivities and specificities for diagnosing pancreaticobiliary cancers. For pancreatic cancer, one meta-analysis evaluated the accuracy of EUS-FNA in diagnosing the correct etiology for a solid pancreatic mass. They reported a sensitivity and specificity of 89% and 96%, respectively [20]. In addition, another study showed that the presence of an onsite cytopathologist during the EUS-FNA was associated with a higher degree of diagnostic accuracy [21]. Needle size in EUS-FNA may also have an effect on diagnostic results. A 2013 meta-analysis of eight studies reported a significant difference in diagnostic sensitivity when using a 25-gauge needle (93%) compared to a 22-gauge needle (85%) [22]. This may be because 25-gauge needles are more flexible and can be easier to handle. However, a more recent meta-analysis did not demonstrate a significant difference between the two in the diagnosis of solid pancreatic lesions [23]. For cholangiocarcinoma, ERCP was once thought to be the gold standard for tissue diagnosis, such as with brush cytology. Many studies have shown that ERCP brush cytology has a specificity approaching 100% for the detection of cholangiocarcinoma, but only a sensitivity of 30% to 50% [24,25,26]. As such, with these lower sensitivities, ERCP may be useful in the diagnostic evaluation if positive, but a negative test cannot rule out malignant disease. On the other hand, tissue sampling with EUS-FNA provides far superior results. Specifically, EUS-FNA was shown to have greater diagnostic sensitivity (94%) and accuracy (94%) than ERCP tissue sampling, while also avoiding biliary tree contamination [27,28]. Sensitivity and specificity for EUS-FNA biopsy of primary ampullary masses are 82% and 100%, respectively [29]. Finally, one meta-analysis of nine studies concluded that EUS-FNA is an accurate and safe method of evaluation for gallbladder masses, with a pooled sensitivity of 84% [30].

Percutaneous biopsy can be performed under ultrasound or CT guidance. Depending on tumor size and operator expertise, the sensitivity and specificity for diagnosing pancreatic cancer range from 80–90% and 98–100%, respectively [31]. There are few studies directly comparing percutaneous and EUS-guided biopsy. One prospective study comparing EUS vs. transabdominal ultrasound or CT guided FNA reported no statistical difference in the diagnosis of pancreatic cancer. However, for lesions less than 3 cm, EUS-FNA may have higher diagnostic accuracy [32,33]. Diagnostic EUS-guided biopsy has also been shown to have substantial cost benefits compared to percutaneous biopsy [34,35]. Perhaps the biggest difference between the two is the risk of disseminating tumor cells intraperitoneally or along the needle path with percutaneous biopsy. In theory, EUS-biopsy is safer, being less likely to cause intraperitoneal spread since the biopsy is performed through the bowel wall and not the skin. In fact, for pancreatic cancer, studies have demonstrated that needle-tract seeding and peritoneal carcinomatosis occur less frequently with EUS-guided biopsy than with percutaneous biopsy [36]. Furthermore, one meta-analysis with 284 patients who underwent EUS-biopsy for biliary and gallbladder masses did not observe any complications of seeding, bile leak, or cholangitis [28]. Similarly, in another study of 101 patients with gallbladder masses who underwent EUS-FNA, there were no complications of procedure-related cholangitis or bile leak in any patient [37]. However, Johnson et al. reported that percutaneous biopsy for pancreatic cancer also did not significantly increase positive peritoneal washings or peritoneal failure rate [38]. Nevertheless, the theoretical risk still exists, so percutaneous biopsy is generally avoided for patients with resectable masses who are candidates for curative surgery,

### 3.2. EUS-FNA vs. FNB

EUS-guided fine needle biopsy (FNB) of a lesion allows core samples to be collected by shearing tissue. This method can obtain larger tissue samples and preserve tissue architecture. Studies comparing EUS-guided FNA and FNB have been mixed. A 2016 meta-analysis found no significant difference between EUS-guided core biopsy needles and standard FNA needles in terms of sample adequacy, diagnostic accuracy, or the acquisition of a core specimen; however, the FNB needle utilized fewer passes [39]. A randomized control trial by Aadam et al. analyzed 140 patients to compare EUS-FNA and EUS-FNB for diagnosing pancreatic masses. Although not statistically significant, the diagnostic yield for FNB was 91.7% compared to 78.4% for FNA [40]. A more recent 2020 meta-analysis of eleven studies reported that for solid pancreatic masses, FNB has superior diagnostic accuracy without compromising safety compared to FNA [41]. With these results, EUS-guided FNB may be a preferable diagnostic approach to FNA, particularly for pancreatic cancer (Table 1).

### 3.3. Genetic Testing

Genetic testing can also be a relevant part of the workup for patients with suspected pancreatic cancer. It is estimated that 5–10% of pancreatic cancer is familial [42], most commonly as part of a genetic predisposition syndrome (such as Peutz–Jeghers or Lynch syndrome) or as “familial pancreatic cancer.” According to the American Society of Clinical Oncology (ASCO), all patients diagnosed with pancreatic adenocarcinoma should undergo assessment of risk for hereditary syndromes, which most importantly includes a comprehensive review of family history [43]. According to the ASCO guidelines, individuals with a family history meeting criteria for familial pancreatic cancer, those with three or more diagnoses of pancreatic cancer in the same side of the family, and those meeting criteria for other genetic syndromes associated with pancreatic cancer are candidates for genetic testing. Testing these at-risk patients can be crucial, as the risk of pancreatic cancer for someone from a “familial pancreatic cancer” family was shown to be nine times higher than for sporadic pancreatic cancer kindreds [44]. Though not a routine part of the assessment for pancreatic cancer, genetic evaluation in the appropriate setting can help identify early disease in high-risk individuals and help to improve outcomes.

## 4. Endoscopic Treatments

Patients with pancreaticobiliary malignancy can develop a variety of complications, such as gastric outlet obstruction, obstructive jaundice, and cholecystitis. Different therapeutic modalities can be used to address each of these complications, including novel interventional EUS procedures such as EUS-guided gastrojejunostomy (EUS-GE), EUS-guided biliary drainage (EUS-BD), and EUS-guided gallbladder drainage (EUS-GLB). In addition, interventional EUS procedures can be utilized for the palliation of unresectable malignancies both for source control with EUS-radiofrequency ablation (EUS-RFA) and for the treatment of abdominal pain refractory to opioid medications with EUS-guided celiac axis neurolysis.

### 4.1. Gastric Outlet Obstruction: EUS-GE vs. Enteral Stent vs. Surgery

**Figure 2:**
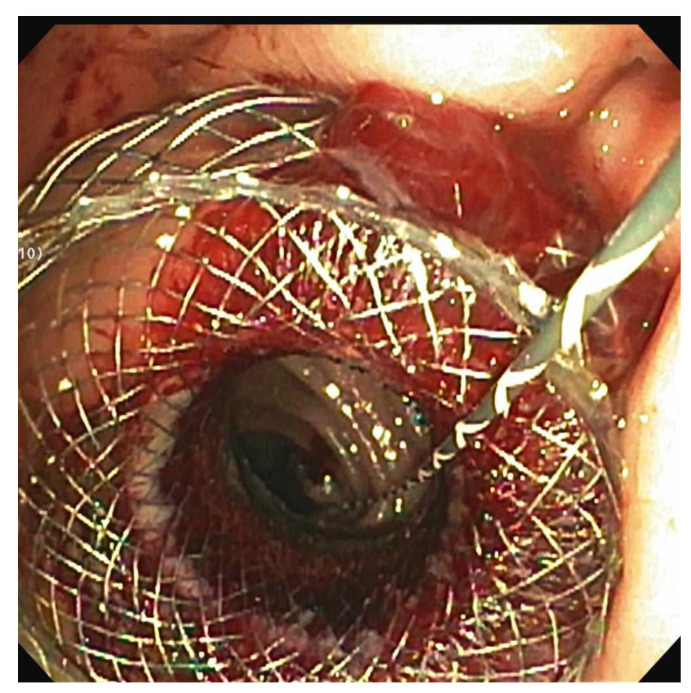
EUS-Gastroenterostomy (EUS-GE): Endoscopic image of a lumen-apposing metal stent deployed between the stomach and the jejunum.

Gastric outlet obstruction (GOO) may present with symptoms of nausea, vomiting and abdominal pain, and patients may suffer from a poor quality of life and poor functional status. Traditionally, treatment options for malignant GOO have consisted of surgical gastrojejunostomy and endoscopic enteral stenting. Surgical gastrojejunostomy is efficacious but invasive, and many patients with malignant GOO are too debilitated to safely undergo surgery. Enteral stent placement with a self-expanding metal stent is efficacious but with limited durability due to inevitable stent ingrowth and re-obstruction, and may not be advisable in patients with longer than 6 months life expectancy [45]. EUS-GE is a novel endoscopic procedure that avoids the morbidity and mortality of surgery and provides long-term durability compared to enteral stenting. This procedure involves the placement of a lumen-apposing metal stent (LAMS) that allows for adequate luminal apposition and the creation of a fistulous tract between the jejunum and stomach (Figure 2).

EUS-GE has now been shown to be both safe and efficacious, with high clinical and technical success rates [46,47,48,49]. Initial studies such as by Tyberg et al. reported the technical success rate to be 92% and clinical success of 85% [46]. Adverse events occurred in three patients (11.5%). A more recent meta-analysis of 297 patients confirmed initial results with pooled technical success, clinical success and complications of EUS-GE of 91%, 88%, and 6.8%, respectively [50]. Ge et al. performed a retrospective study comparing EUS-guided gastroenterostomy (EUS-GE) to enteral stent placement and found the technical success to be 100% in both groups. However, they found higher clinical success in the EUS-GE group of 95.8% compared with 76.3% in the enteral stent group, decreased adverse events (20.8% vs. 40.2%) and better stent function [51]^.^ EUS-GE had higher rates of initial clinical success and lower rates of stent failure requiring repeat intervention. Enteral stenting was associated with almost 13-fold odds of reintervention when compared to EUS-GE. Khashab et al. conducted an international, multicenter study for patients with malignant GOO, comparing EUS-GE with surgical gastrojejunostomy (SGJ) [52]. They included 93 total patients, of which 30 received EUS-GE. They found the rate of adverse events to be lower in the EUS-GE group of 16% compared with 25% in the surgical group, but the results were not statistically significant (*p* = 0.3). The mean length of hospital stay was similar and the time to reintervention was similar between the two groups. Additional studies comparing EUS-GE with laparoscopic-GJ (Lap-GJ) have shown endoscopy to be associated with decreased adverse events of 12% compared with 41% [53]. EUS-GE was also shown to have significant cost savings, being about one third of the cost when compared to Lap-GJ [54]. Stent migration or misdemployment has been reported during EUS-GE due to the technical complexity of this procedure. This happens when one flange of the LAMS is deployed outside the lumen of the small bowel (distal flange misdeployment) or outside the lumen of the stomach (proximal flange misdeployment). If this occurs peri-procedurally, often the procedure can be salvaged by bridging the LAMS with an additional stent [51,52]. Alternatively, in the case of distal flange misdeployment, the LAMS can be removed and the defect closed with endoscopic suturing or over-the-scope clips.

Overall, the choice of treatment of GOO is dependent on patient comorbidities and overall life expectancy as well as available endoscopic expertise at a given center. Patients with less than six months life expectancy or who require short-term relief prior to surgical resection may benefit most from enteral stent placement; those with a need for longer duration of obstructive relief may benefit from EUS-GE. However, EUS-GE is technically complex and must be performed by expert endoscopists with expertise in therapeutic endoscopic ultrasound, and thus should only be offered in expert centers.

### 4.2. Biliary Obstruction: ERCP vs. EUS-BD

**Figure 3:**
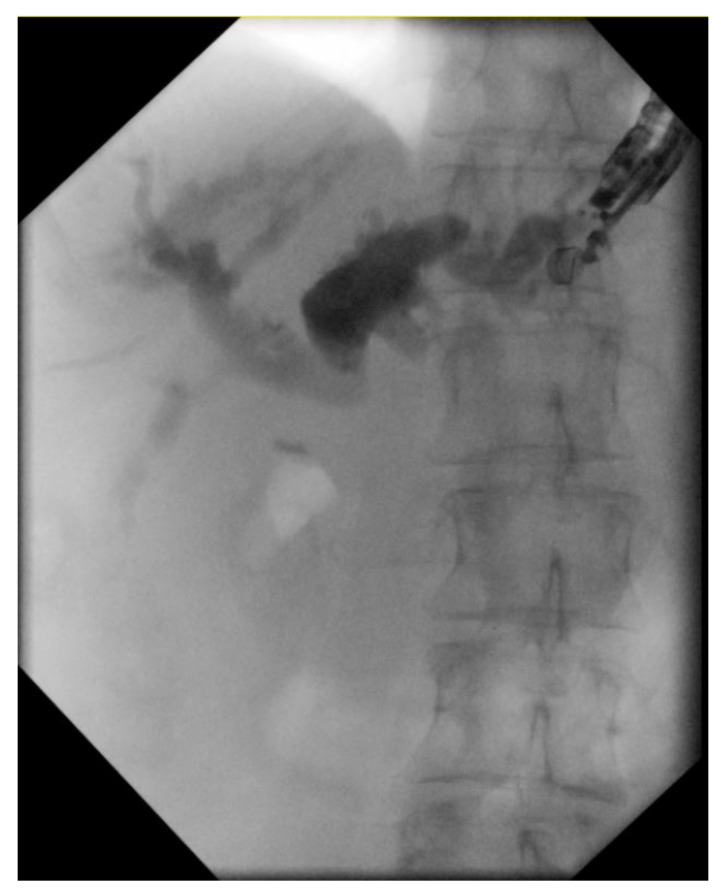
EUS-Biliary Drainage (EUS-BD): Fluoroscopic image of an EUS-obtained cholangiogram.

Endoscopic retrograde cholangiopancreatography (ERCP) is currently the gold standard to relieve malignant biliary obstruction (MBO). However, limitations such as surgically altered anatomy, periampullary diverticuli, and large tumors obstructing the gastrointestinal lumen or the ampulla itself make ERCP impossible in certain patients [55]. In addition to ERCP, percutaneous transhepatic biliary drainage (PTBD) or surgical biliary bypass can be offered to patients. However, PTBD is associated with discomfort from percutaneous drains, high complication rates and the need for reinterventions [56]. Endoscopic ultrasound-guided biliary drainage (EUS-BD) has been developed as an alternative procedure for biliary drainage in MBO (Figure 3). Current studies have shown EUS-BD to have better clinical success, fewer adverse events and lower reintervention rates when compared to PTBD and non-inferior in terms of safety and efficacy when compared to conventional ERCP [57,58].

Since EUS-BD was first described by Giovannini et al. in 2001, many studies have been performed to validate its use [59]. Multicenter studies have shown the technical success and efficacy to range from 93–100%, and a meta-analysis from 2016 conducted by Khan et al. reported the technical success of EUS-BD to be 90% [58,60]. A recently published systematic review and meta-analysis compared EUS-BD with ERCP and showed similar overall adverse events rates; however, procedure-related pancreatitis was 0% for the EUS-BD group and 8.7% for the ERCP group [61]. The incidence of reintervention for EUS-BD was 19.4% compared with 25.9% in ERCP. Further, EUS had a tendency toward lower reintervention rates and longer stent patency, although it was not statistically significant. Bile leakage was found to be the most commonly reported adverse event in EUS. Initially, EUS-BD was used as a second line therapy when ERCP was unsuccessful or not feasible. However, recent studies have demonstrated EUS-BD to have a role in the primary palliation of MBO when compared to ERCP. Paik et al. conducted a multicenter, randomized clinical trial including 125 patients comparing EUS-BD to ERCP for primary palliation in MBO. Technical and clinical success was similar for both procedures, but EUS-BD was associated with a lower rate of adverse events (6.3% vs. 19.7%, *p* = 0.03), including post-procedure pancreatitis (0 vs. 14.8%), a lower reintervention rate (15.6% vs. 42.6%), and a higher rate of stent patency (85.1% vs. 48.9%). EUS-BD was also associated with more preserved quality of life than transpapillary stenting [62].

Overall, biliary decompression can be critical in patients with pancreaticobiliary malignancy for the relief of symptoms, the prevention of cholangitis, and in some cases, preparation for surgical resection. The choice of decompression technique must be based on the patient’s anatomy/access to the ampulla, treatment plan (surgical resection vs. palliation), and available expertise. It is important for EUS-BD to be performed by expert endoscopists at tertiary care centers with experience in therapeutic EUS, but growing experience and training of this procedure will push EUS forward as a reliable alternative to ERCP and other modalities for MBO.

### 4.3. Cholecystitis: EUS-GLB vs. Percutaneous Drainage

For resectable pancreaticobiliary malignancies, the gallbladder is generally removed at the time of surgery. However, for patients who are non-operative or who require neoadjuvant therapy prior to surgical resection, acute cholecystitis can occur from malignant obstruction of the cystic duct. Non-operative options for the treatment of acute cholecystitis are percutaneous cholecystostomy tube placement (PT-GLB), endoscopic transpapillary gallbladder drainage (ET-GLB), and EUS-guided gallbladder drainage (EUS-GLB).

**Figure 4:**
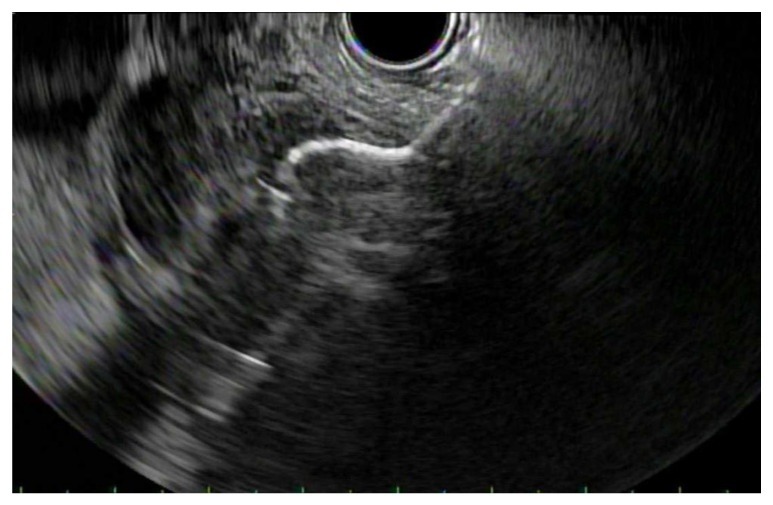
EUS-Gallbladder Drainage (EUS-GLB): Endosonographic image of a lumen-apposing metal stent being deployed between the gallbladder and the stomach.

Recently, EUS-GLB has emerged as a safe and effective alternative to traditional modalities such as percutaneous drainage and endoscopic retrograde cholangio-pancreatography (Figure 4) [63]. Age and medical comorbidities limit surgery for these patients, and therefore non-surgical options are increasingly utilized. Limitations of percutaneous drainage include indwelling catheter, pain at the insertion site and decreased quality of life [64]. Further, complications of percutaneous drainage include risks of bleeding, bile site leakage and recurrent cholecystitis, which has ranged from 22–47% [65]. Studies have shown the clinical success rate for EUS-GLB to be similar to percutaneous drainage [55]. A retrospective, international, multicenter analysis found EUS-GLB to have significantly lower overall adverse events, decreased length of hospital stay and unplanned admissions compared to PT-GLB [66]. Two recent meta-analyses evaluating EUS-GLB vs. PT-GLB found better clinical success in the EUS-GLB group and lower rates of post-procedure adverse events, shorter hospital stays, fewer reinterventions and readmissions in the EUS-GLD group [67,68]. Recently, a randomized control study of 80 patients comparing EUS-GBD to PT-GBD found that the two groups had similar efficacy, but the EUS-GBD groups had significantly less immediate and long-term (1 year) adverse events, reintervention rate, unplanned admissions, and recurrent cholecystitis [69]. Higa et al. performed a retrospective study of 78 patients comparing EUS-GLB and ET-GBD. They found clinical success to be higher with 95% in EUS versus 76.3% in transpapillary drainage. They did not find a difference in outcomes for the duration of follow-up, reintervention rates, hospital length of stay, and overall adverse events [70]. A systematic review and meta-analysis comparing these two endoscopic procedures included five studies and 857 total patients in high-risk surgical patients. EUS-GBD has a higher rate of technical and clinical success compared to ET-GBD. While the rates of overall adverse events were statistically similar, EUS-GBD has a lower rate of recurrent cholecystitis [71].

Overall, cholecystitis in pancreaticobiliary malignancies can be treated with percutaneous or endoscopic drainage. However, growing evidence suggests that with the lower rates of adverse events, reinterventions, unplanned admissions, and recurrent cholecystitis, EUS-GLB should be considered as a therapeutic option of choice.

### 4.4. Palliation for Source Control: EUS-RFA

**Figure 5:**
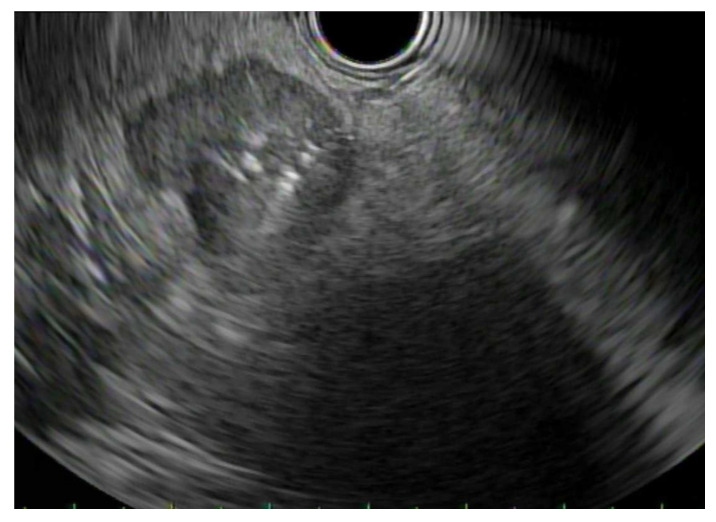
EUS-Radiofrequency Ablation (EUS-RFA): Endosonographic image of a pancreatic lesion being ablated by EUS-RFA.

Radiofrequency ablation (RFA) is a local ablative technique that has been used for the treatment of different solid tumors, including lung, liver, bone and prostate. It uses heat to destroy tumor cells by thermal coagulation and protein denaturation [72]. RFA can be administered in the pancreaticobiliary tree via EUS into solid pancreatic masses or directly to the biliary mucosa during ERCP (Figure 5). In patients with unresectable pancreatic tumors on systemic therapy, EUS-RFA can be used as an adjuvant therapy for source control.

EUS provides excellent visualization, real-time imaging guidance and the localization of local structures and vasculature to allow for safe tissue ablation. Goldberg et al. first reported the use of EUS-RFA of the normal pancreas in pigs in 1999. They documented minimal complications of transmural gastric burns, serosal small intestinal burns, and elevated lipase with focal pancreatitis, without any major complications noted [73]. Gaidhane et al. evaluated EUS-RFA of the pancreatic head using a Habib EUS-RFA catheter in pigs, which was well tolerated with a minimum amount of pancreatitis [74]. In human studies, EUS-RFA has been most widely studied in pancreatic neuroendocrine tumors. A recently published systematic review and meta-analysis of 13 studies including 134 patients reported the technical success rate to be 100% and clinical success to be 92%. The pooled overall adverse event rates were 15%, with abdominal pain being the most reported at 9.8% [75].

Limited data exist of EUS-RFA for unresectable pancreatic adenocarcinomas. Choi et al. reported a prospective study of EUS RFA in benign solid pancreatic tumors with a technical success rate of 100%. Radiologic complete remission was achieved in 70%, and the median diameter changed from 20 to 6.5 mm (58.9% reduction in the lesion). Abdominal pain and pancreatitis were the only adverse events reported [76]. Thosani et al. reported EUS-RFA efficacy in a multicenter retrospective study which included 21 patients, 47% of which had pancreatic ductal adenocarcinoma [77]. Alvarez-Sanchez et al. reported a review of 42 patients who underwent EUS-RFA for a variety of pancreatic lesions, 28 of which were unresectable pancreatic cancer. Technical success was achieved in 36 patients (86%). There was no procedure-related mortality, mild early complications were observed in 9 of 36 patients (25%), and there was only one case of mild acute pancreatitis [78].

Prospective and controlled studies are needed with larger sample sizes and follow-up to better establish the safety and long-term efficacy of EUS-RFA. However, it is an exciting potential adjuvant palliative treatment for patients with unresectable pancreaticobiliary malignancies, or in the future as a downstaging therapy for borderline resectable patients.

### 4.5. Palliation for Abdominal Pain: Celiac Neurolysis

**Figure 6:**
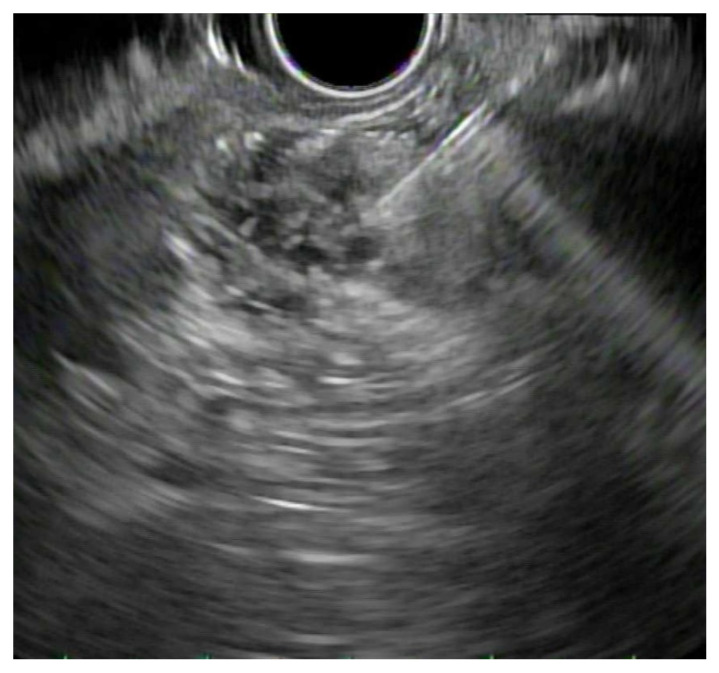
EUS Celiac Plexus Neurolysis (CPN): Endosonographic image of a celiac plexus neurolysis.

Pain control in patients with pancreaticobiliary malignancies can be a challenge for physicians. Traditional therapy with non-opioid analgesics may not provide appropriate care, and adverse events of opioids such as dependence and addiction can limit the use of these medications. Celiac plexus neurolysis (CPN) is a well-established technique for the control of abdominal pain in patients with pancreaticobiliary cancer. The celiac plexus is located around the celiac axis and contains several ganglia that transmit pain sensations from upper abdominal organs such as the pancreas, gallbladder and liver. CPN uses alcohol as a neurolytic agent to ablate the celiac plexus nerves and reduce narcotic requirements in these patients. Endoscopic ultrasound has emerged as a favorable approach when compared to intraoperative or radiographic treatment of CPN (Figure 6).

While CPN has traditionally been used as late in the disease course for pain control, studies have suggested benefits for early use during the time of diagnosis [79]. Early use of EUS-CPN can be incorporated as a treatment option during endoscopic interventions such as fine needle aspiration. Wyse et al. performed a randomized, double-blinded control trial to compare the early use of endoscopic ultrasound delivery of CPN compared with conventional pain management [79]. They found that early EUS-CPN provided better pain control at 1 and 3 months and may moderate morphine consumption when compared to controls. A systematic review and meta-analysis gathered eight studies including 283 patients and reported pain relief in 80% of patients who have undergone EUS-guided CPN for pancreatic cancer [80]. Studies have demonstrated overall improvements in pain relief, a reduced need for opioid use and fewer opioid-related side effects [81]. While CPN has been shown to be safe and effective, reported common complications are related to the blockade of sympathetic efferent activity. Transient diarrhea and hypotension were the most frequently reported adverse events. Transient increases in pain or exacerbations were reported in 4% of patients [82]. Very few serious adverse events have been reported, but these include retroperitoneal bleeding, vascular injury or ischemic complications due to iatrogenic injury during injection and spinal cord injury.

EUS-CPN is a safe and effective method for treating abdominal pain in patients with pancreatic cancer; however, this procedure should be reserved for unresectable tumors as the effects are irreversible.

## 5. Conclusions

Endoscopic ultrasound is an integral part of the diagnosis and treatment of pancreaticobiliary malignancies and their complications. EUS is critical in the diagnosis and staging of cancers, and recent advances in EUS have led to the development of novel therapeutic EUS-guided procedures to treat complications and provide adjuvant therapy. However, the tailoring of procedures must be individualized based on patient-specific and disease-specific factors.

## Figures and Tables

**Table 1:** Outcomes of EUS-guided FNA vs. fine needle biopsy (FNB).

Study	Study Type	Total Participants in Study	Diagnostic Accuracy (%)		Mean Number of Passes Required for Adequate Diagnosis		Significant Difference in Adverse Events?
			FNA	FNB	FNA	FNB	
Bang et al. (2016)	Meta-analysis	576	85.8	86.2	2.8 *	1.6 *	No
Aadam et al. (2016)	RCT **	140	67.1 *	90.0 *	3.0	2.8	No
Cheng et al. (2018)	RCT	408	80.0 *	91.4 *	-	-	No
Riet et al. (2019)	RCT	608	78.0 *	87.0 *	-	-	No
Renelus et al. (2020)	Meta-analysis	1365	81.0 *	87.0 *	2.3 *	1.6 *	No

Bold * indicates statistical significance between FNA and FNB. ** RCT = randomized control trial
